# The synergistic potential of combining PD-1/PD-L1 inhibitors with conventional therapies in esophageal squamous cell carcinoma

**DOI:** 10.3389/fphar.2026.1894567

**Published:** 2026-10-16

**Authors:** Ruyan Li, Zihao Heng, Xi Cheng

**Affiliations:** 1 School of Health Medicine, Nantong Institute of Technology, Nantong, China; 2 Department of Gynecology and Obstetrics, Affiliated Hospital of Nantong University, Medical School of Nantong University, Nantong, China

**Keywords:** combination therapy, conventional therapy, esophageal squamous cell carcinoma, PD-1 inhibitor, PD-L1 inhibitor

## Abstract

Esophageal squamous cell carcinoma (ESCC) is associated with a poor prognosis, and the efficacy of conventional therapies (chemotherapy, radiotherapy, and surgery) remains limited. The advent of programmed death 1 (PD-1)/programmed death-ligand 1 (PD-L1) inhibitors has substantially altered the treatment paradigm for ESCC. The combination of these immune checkpoint inhibitors (ICIs) with conventional treatments demonstrates significant synergistic potential. Standard oncological regimens often center on chemotherapy, radiotherapy, surgery, or various combinations thereof. This synergy stems from complementary mechanisms: conventional therapies can induce immunogenic cell death, release tumor antigens, and remodel the immunosuppressive tumor microenvironment, thereby laying the groundwork for immunotherapy. In turn, PD-1/PD-L1 inhibitors work by releasing the “brakes” on T cells, activating and enhancing the host anti-tumor immune response. This facilitates the elimination of residual cancer cells and establishes long-term immune surveillance. In conclusion, the combination of PD-1/PD-L1 inhibitors with conventional therapy achieves a greater-than-additive (“1 + 1>2”) effect and has evolved into a standard first-line treatment strategy for advanced/metastatic ESCC, substantially improving patient outcomes. Future research will focus on optimizing combination protocols, overcoming resistance, and precisely identifying patient populations most likely to benefit.

## 1 Review methodology

This is a narrative review that systematically synthesizes the current literature on programmed death 1 (PD-1)/programmed death-ligand 1 (PD-L1) inhibitors combined with conventional therapies for esophageal squamous cell carcinoma (ESCC). A comprehensive literature search was conducted in PubMed, Web of Science, Cochrane Library, and CNKI (China National Knowledge Infrastructure) from database inception to March 2026. The search terms included: (“esophageal squamous cell carcinoma” OR “ESCC”) AND (“PD-1” OR “PD-L1” OR “immune checkpoint inhibitor” OR “pembrolizumab” OR “nivolumab” OR “camrelizumab” OR “sintilimab” OR “toripalimab” OR “tislelizumab”) AND (“chemotherapy” OR “radiotherapy” OR “surgery” OR “neoadjuvant” OR “adjuvant” OR “combination”).

## 2 Epidemiology and clinical landscape of esophageal squamous cell carcinoma

Esophageal cancer (EC) is a common malignant tumor that poses a significant threat to global health, characterized by its high malignancy and refractory nature. Investigations into its risk factors reveal a distinct etiology for ESCC in the Chinese population: its occurrence shows a less pronounced association with smoking and alcohol consumption compared to Western populations. Instead, it is closely linked to a positive family history, nutritional deficiencies, poor lifestyle habits, and specific dietary practices. Furthermore, genetic polymorphisms may also contribute to the high incidence of EC in this region (Zhu et al., 2023). Early-stage ESCC is often clinically silent or presents with nonspecific symptoms, making detection challenging. Common early manifestations include a vague sensation of a foreign body in the esophagus, mild dysphagia with hard foods, or a burning sensation behind the sternum. However, by the time patients develop prominent symptoms such as significant dysphagia or palpable cervical lymphadenopathy, the disease has typically progressed to an intermediate or advanced stage. Consequently, nearly half of all patients are diagnosed at a point where they have missed the optimal window for curative surgical intervention. In late stages, symptoms of distant metastasis may appear, including cough, hemoptysis, and widespread lymphadenopathy (Moehler et al., 2020).

### 2.1 Incidence, mortality, and geographical distribution

Esophageal squamous cell carcinoma is a prevalent subtype of EC, accounting for approximately 80%–90% of all cases. It is characterized by high aggressiveness, a poor prognosis, and significant geographical clustering, being primarily prevalent in East Asia (China, Mongolia), Central Asia, along the East African Rift Valley, and in Southern Africa (Abnet et al., 2018). Notably, China bears more than half of the global ESCC burden. Compounding this high incidence is the challenge of late diagnosis, as the disease often presents with insidious onset. Consequently, about 70% of patients are identified at an intermediate or advanced stage, missing the window for potentially curative surgery, which directly results in a poor 5-year overall survival rate of merely 10%–20% (Zhu et al., 2023; Mattiuzzi and Lippi, 2019). Thus, patients with ESCC face a low 5-year survival rate and a generally poor prognosis. However, despite certain advances in conventional therapies such as surgery, chemotherapy, and radiotherapy, the survival rate for patients with advanced disease remains low. According to the 2022 global cancer statistics, there were 510,000 new cases of EC worldwide, ranking 11th among all malignancies; there were 450,000 deaths, ranking seventh, indicating a relatively high mortality risk. New cases and deaths from EC in China each account for approximately 40% of the global total, making it the country with the heaviest burden of EC worldwide (Bray et al., 2024). Globally, over half of all new EC cases occur in China, with high-incidence areas primarily concentrated in the Taihang Mountain region. It is not only the sixth most common malignant tumor in China but also the fourth leading cause of cancer-related death. ESCC is the predominant histological subtype of EC, accounting for approximately 90% of all EC cases (Zhao et al., 2024).

### 2.2 Etiology and pathogenesis

Tobacco smoking and alcohol consumption are internationally recognized as two major independent risk factors for ESCC. Multiple carcinogens in tobacco, such as nitrosamines and polycyclic aromatic hydrocarbons, can directly damage the esophageal mucosa. Alcohol, particularly spirits, acts as a solvent that facilitates the absorption of these carcinogens. Furthermore, acetaldehyde, a metabolite of ethanol, is itself a recognized carcinogen. Notably, tobacco and alcohol exhibit a significant synergistic carcinogenic effect. Poor lifestyle habits also contribute to the etiology. The chronic consumption of excessively hot beverages, such as tea, soup, or porridge, can repeatedly scald the esophageal mucosa. This repeated thermal injury leads to chronic inflammation and hyperplasia, creating a tissue microenvironment conducive to malignant transformation.

The pathogenesis of ESCC is a complex process involving the dysregulation of multiple molecular pathways, reprogramming of cellular metabolism, remodeling of the tumor microenvironment, and immune evasion. Genetic alterations in specific genes, such as *TP53* (encoding the p53 protein), disrupt critical cellular control mechanisms. The *TP53* tumor suppressor gene is the most frequently mutated gene in ESCC. Its functional loss impairs key cellular processes including DNA damage repair, cell cycle regulation, and the induction of apoptosis (programmed cell death), thereby facilitating uncontrolled cell proliferation and tumorigenesis (Case and Domann, 2014). Beyond singular gene defects, the aberrant activation of entire signal transduction pathways is commonplace. When esophageal squamous epithelial cells are subjected to chronic stimuli (e.g., from the risk factors previously described), these internal signaling cascades—such as the phosphatidylinositol 3-kinase (PI3K)/AKT, Nuclear Factor-kappa B (NF-κB), and NOTCH pathways—can become constitutively active. This disrupts the delicate balance between cellular proliferation and apoptosis, pushing cells towards relentless and unlimited division.

Finally, these evolving tumor cells exploit and shape a permissive tumor microenvironment. A critical aspect of this is the induction of immune dysfunction. Tumor cells develop mechanisms to evade immune surveillance, such as upregulating immune checkpoint molecules. This impairs the immune system’s capacity to recognize and eliminate abnormal cells, allowing the tumor to thrive, persist, and metastasize.

## 3 Conventional treatment modalities

The traditional treatment paradigm for ESCC primarily rests on a triad of surgery, radiotherapy, and chemotherapy. A multidisciplinary comprehensive treatment strategy is employed, tailored according to factors such as tumor stage, location, and the patient’s overall physical condition.

### 3.1 Surgery

For resectable, early-stage (Stage I/II) ESCC, radical surgical resection remains the primary treatment of choice for most patients. The goal is the complete removal of the primary tumor along with regional lymph nodes. The standard surgical approaches include the minimally invasive McKeown esophagectomy (a three-incision procedure involving the right chest, upper abdomen, and left neck) or the Ivor-Lewis esophagectomy (a two-incision procedure involving the right chest and abdomen). As the cornerstone of early-stage ESCC treatment, surgical intervention has been a well-established modality for over a century (Watanabe et al., 2020).

### 3.2 Radiotherapy

For locally advanced (Stage III) yet resectable tumors, neoadjuvant chemoradiotherapy (NCRT) followed by surgery remains the standard treatment regimen (Category 1 recommendation) (Kato et al., 2024). Studies have confirmed that, compared to surgery alone, preoperative taxane/platinum-based concurrent chemoradiotherapy can significantly downstage the tumor, increase the R0 resection rate, and improve patients’ overall survival.

### 3.3 Chemotherapy

However, previous studies on high-dose radiotherapy combined with cisplatin and fluorouracil indicate that increasing the radiation dose leads to elevated toxicity and poorer patient treatment compliance. Furthermore, over 50% of patients experience local or distant recurrence after definitive concurrent chemoradiotherapy (CCRT), with a 1-year progression-free survival (PFS) rate of 40%–66%. Consequently, there is an urgent need for more effective and less toxic treatment strategies for patients with unresectable locally advanced ESCC. For unresectable locally advanced, recurrent, or metastatic ESCC, treatment primarily relies on systemic chemotherapy and palliative radiotherapy, with the goals of controlling disease progression, alleviating symptoms (such as dysphagia and pain), and prolonging survival. The traditional standard chemotherapy regimens consist of two-drug combinations, typically a platinum-based agent (e.g., cisplatin, nedaplatin) paired with either a fluorouracil analog (5-FU or S-1) or a taxane (paclitaxel, docetaxel). For patients in poor general condition, single-agent chemotherapy may also be considered. At this stage, radiotherapy is mainly used to address complications caused by local lesions, such as obstruction, bleeding, or bone metastasis pain. Precise radiotherapy techniques (e.g., intensity-modulated radiotherapy) are employed to better protect surrounding normal organs while reducing adverse effects. In China, the frequently used first-line chemotherapy regimen of paclitaxel combined with cisplatin has demonstrated an objective response rate (ORR) of 62.1% and a median PFS of 5.6 s in patients with advanced ESCC (Luo et al., 2021).

## 4 Novel therapeutic approaches for esophageal squamous cell carcinoma

In recent years, the field of novel therapies for ESCC has witnessed significant progress, demonstrating considerable potential, particularly in the areas of immunotherapy, targeted therapy, and other innovative therapeutic strategies.

### 4.1 Immunotherapy

In the realm of immunotherapy, PD-1 inhibitors (such as pembrolizumab, sintilimab, and camrelizumab) have emerged as the standard of care for second-line treatment of advanced ESCC and are progressively extending into first-line and perioperative treatment settings (Doi et al., 2018).

Pivotal studies such as KEYNOTE-590 have confirmed that pembrolizumab in combination with chemotherapy significantly extends both overall survival and PFS in patients with advanced disease (Sun et al., 2021). Furthermore, neoadjuvant immunochemotherapy (NICT) has demonstrated promising efficacy in resectable locally advanced ESCC, achieving a pathological complete response (pCR) rate of up to 25%. Compared to traditional neoadjuvant chemoradiotherapy, NICT is associated with a significant reduction in surgical complications such as recurrent laryngeal nerve injury, while yielding an encouraging 2-year survival rate of 76.2% (Wang X. et al., 2025). A separate clinical study analyzing treatment data for cervical EC patients from 1989 to 2014 found that the adoption of concurrent chemoradiotherapy increased from 1% to 49%, accompanied by an extension in overall survival time. This finding affirms the clinical value of concurrent chemoradiotherapy in EC management (Pardoll, 2012). When directly compared, patients receiving NCRT demonstrated inferior outcomes to those receiving NICT in terms of pCR rate, tumor regression grade, lymph node negativity rate, and the incidence of neural invasion and vascular tumor thrombus. However, overall survival rates were similar between the NICT and NCRT groups (Qin et al., 2024). It is crucial to note that immune-related adverse events (irAEs), such as interstitial pneumonia and myocarditis, require careful monitoring and timely intervention. This is particularly important for elderly patients, those who have undergone multiple lines of therapy, or individuals with underlying comorbidities.

### 4.2 Targeted therapy

Research in targeted therapy focuses on the specific molecular mechanisms of ESCC. A team discovered that the FAK/SFK tyrosine kinase complex drives metastatic proliferation and adaptive survival of metastases by phosphorylating the metabolic enzymes ACLY (at Tyr542/652) and ALDOA (at Tyr174/302/328) (Chen et al., 2025). Targeting this axis, the natural compound quercetagitrin specifically inhibits ALDOA phosphorylation. In animal models, it reduced lymph node metastases by 75%, and demonstrated a synergistic effect when combined with FAK/SFK inhibitors such as defactinib (Chen et al., 2025). Additionally, other targeting strategies are emerging. One involves targeting RNA modification: The AT10-mediated ac4C RNA modification enhances the translation of ATP6V0E1 mRNA, promoting lysosomal acidification and tumor metastasis. Its small-molecule inhibitor, G-749, has shown anti-metastatic potential in preclinical studies (Zhan et al., 2025).

Beyond the FAK/SFK axis, several other molecular targets are under active investigation in ESCC (Jubashi et al., 2024); Human Epidermal Growth Factor Receptor 2 (HER2) amplification, although more commonly observed in adenocarcinoma of the gastroesophageal junction, is also detected in a subset of ESCC cases and has been therapeutically explored with trastuzumab or antibody-drug conjugates such as trastuzumab deruxtecan (HERES Trial, 2021); however, its efficacy appears less definitive than in HER2-positive gastric cancer. Fibroblast growth factor receptor (FGFR) alterations, including *FGFR1* and *FGFR2* amplifications, are present in approximately 10%–20% of ESCC cases and have been targeted in early-phase trials with FGFR inhibitors such as erdafitinib and futibatinib, though the observed activity remains modest (ClinicalTrials.gov, 2021). Aberrant activation of the PI3K/AKT/mTOR pathway is frequently driven by *PIK3CA* mutations or Phosphatase and tensin homolog deleted on chromosome ten *(PTEN)* loss, and inhibitors targeting this axis, including alpelisib and everolimus, are under evaluation, albeit with limited single-agent activity (Luo et al., 2022). Notably, approximately 10%–22% of ESCC cases harbor Nuclear Factor Erythroid 2-Related Factor 2 *(NRF2)* mutations, which can aberrantly activate the PI3K pathway as a downstream effector (Subramaniyan et al., 2026), providing a rationale for combined *NRF2* and PI3K pathway inhibition as a novel therapeutic strategy. Furthermore, Trophoblast Cell Surface Antigen 2 (TROP2) is overexpressed in over 94% of ESCC patients, and TROP2-targeting antibody-drug conjugates, such as sacituzumab govitecan, have demonstrated synergistic anti-tumor effects in preclinical models when combined with oxidative phosphorylation inhibitors (Liu et al., 2025). Nevertheless, most targeted agents have yet to demonstrate survival benefits comparable to those of PD-1 inhibitors, and their greatest value may lie in biomarker-selected subgroups or in combination with immunotherapy. Ongoing biomarker-driven trials are expected to clarify the optimal integration of targeted therapies into the ESCC treatment algorithm.

### 4.3 Other novel therapeutic strategies

Other novel therapeutic approaches focus on modulating the tumor immune microenvironment (TIME). The biological roles and significance of TIME across various tumor types are increasingly being recognized (Kudo et al., 2017; Zhang et al., 2025). TIME refers to the cellular and molecular composition surrounding a tumor, encompassing immune cells, stromal cells, vascular cells, the extracellular matrix, and diverse signaling molecules, constituting a dynamic ecosystem. For instance, targeting *ISCU* expression in tumor-associated macrophages (TAMs) can reverse their M2 polarization, thereby enhancing the efficacy of anti-PD-1 therapy. Inhibiting the Nicotinamide N-methyltransferase *(NNMT)* activity in cancer-associated fibroblasts (CAFs) can alleviate immunosuppression and boost the function of CD8^+^ T cells (Li L. et al., 2025). Additionally, aptamer-based nanosystems specific to EC (e.g., EA1-EYLNs-PTX/siEFNA1), which improve tumor targeting and gene silencing efficiency, offer promising new avenues for combination therapy (Xie et al., 2024).

## 5 Mechanism of the PD-1/PD-L1 pathway and immune checkpoint inhibitors

The treatment of ESCC has entered an era of multidisciplinary comprehensive therapy, where combination strategies have become mainstream. The core of these strategies lies in the integration of novel therapeutic approaches (such as targeted therapy and immunotherapy) with conventional methods (including surgery, radiotherapy, and chemotherapy).

Among the various novel therapies, the suppression and reprogramming of the immune system play a pivotal role (Wang D. R. et al., 2022). The PD-1/PD-L1 pathway is a central focus in tumor immunotherapy. PD-1 inhibitors, key representatives of immunotherapy, operate on the principle of “targeting immunity.” Their mechanism involves specific monoclonal antibodies blocking the interaction between PD-L1 on tumor cells and PD-1 on T cells. This blockade releases the inhibitory brakes on T cells within the tumor microenvironment, thereby reactivating the body’s own immune system to recognize and attack cancer cells (Iwasaki et al., 2025; Huo et al., 2024). PD-1, a member of the CD28 receptor family, is a co-inhibitory receptor and a key immune checkpoint protein. It is primarily expressed on the surface of activated T cells, B cells, and macrophages. Its ligand, PD-L1, is highly expressed on many cancer cells (Tanaka et al., 2017; Han et al., 2020), but shows low expression in normal tissues (e.g., placenta, lung, liver). The binding of PD-L1 to PD-1 transmits a negative regulatory signal. This signal inhibits the proliferation of activated T cells, induces T cell exhaustion, suppresses the overall immune response, and ultimately enables tumor cells to evade immune surveillance (Hatogai et al., 2016). Figure 1 illustrates the role of the PD-1/PD-L1 pathway in tumor immune evasion and the therapeutic mechanism of anti-PD-1 antibodies.

**FIGURE 1:**
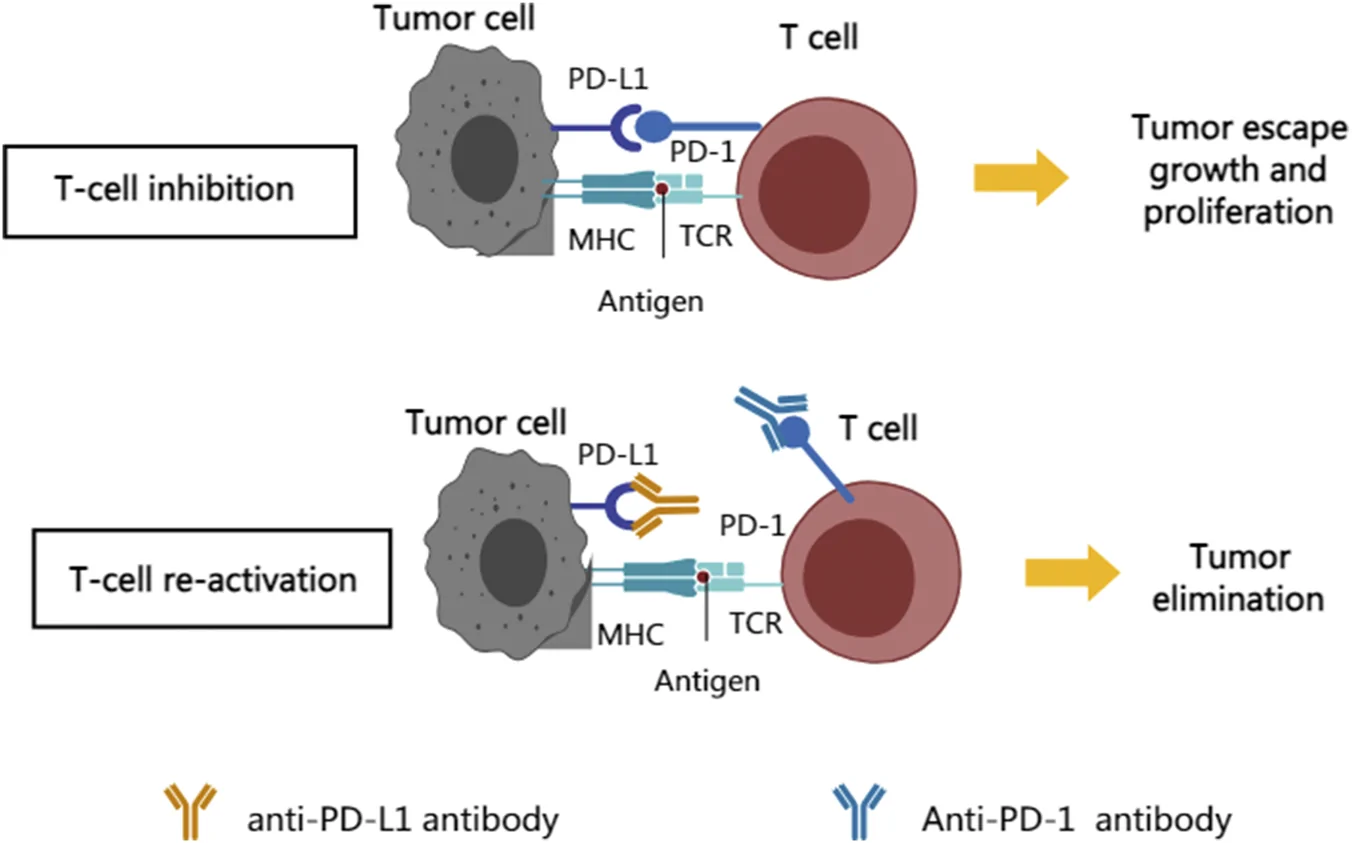
The role of the PD-1/PD-L1 pathway in tumor immune evasion and the therapeutic mechanism of anti-PD-1 antibodies.

In various malignancies (such as melanoma and lung cancer), tumor cells exploit the PD-1/PD-L1 pathway to evade immune destruction. Genetic mutations or inflammatory signals (e.g., interferon-γ, IFN-γ) can induce the overexpression of PD-L1 on the tumor cell surface, forming an immunosuppressive “shield.” Within the tumor microenvironment, PD-L1 (which may be expressed by tumor cells or infiltrating immune cells) binds to PD-1 on the surface of T cells. This interaction triggers downstream inhibitory signals, suppressing the immune effector functions of T lymphocytes and thereby facilitating tumor immune escape (Kudo et al., 2017). PD-1 inhibitors and PD-L1 inhibitors are crucial classes of immune checkpoint inhibitors immune checkpoint inhibitors (ICIs) that counteract this evasion mechanism. PD-1 inhibitors target the PD-1 receptor on T cells. The Fab region of a monoclonal antibody binds to the extracellular domain of PD-1, creating a steric hindrance that prevents its interaction with its ligands, PD-L1 and PD-L2. PD-L1 inhibitors target the PD-L1 molecule on tumor or immune cells. They block the engagement between PD-L1 and PD-1, and also prevent PD-L1 from binding to another receptor, B7-1 (CD80). By precisely blocking this “brake” signal, PD-1/PD-L1 inhibitors reinvigorate T-cell function and remodel the tumor microenvironment, synergistically activating anti-tumor immunity at molecular, cellular, and systemic levels. Therefore, blocking the PD-1/PD-L1 signaling pathway to reactivate T-cell cytotoxicity and induce tumor regression is a validated therapeutic strategy (Alsaab et al., 2017).

Capitalizing on this mechanism, PD-1 inhibitors (e.g., pembrolizumab, sintilimab, nivolumab) can be flexibly combined with various conventional therapies. Clinically, PD-1 inhibitors combined with chemotherapy represent the most common approach. Chemotherapy not only directly kills tumor cells and reduces tumor burden but also can induce immunogenic cell death (ICD), further priming anti-tumor immune responses. PD-1 inhibitors combined with radiotherapy leverage the “abscopal effect” of radiation and its ability to modulate the tumor microenvironment, enhancing systemic anti-tumor efficacy (Iwasaki et al., 2025). In the perioperative setting, the combination of PD-1 inhibitors with chemotherapy and surgery is being actively explored and optimized to improve patient outcomes (Xu et al., 2018). The broad application and ongoing investigation of these combination strategies lay the foundation for the following in-depth discussion of specific regimens and their clinical evidence.

## 6 PD-1 inhibitors in combination with conventional therapies

The combination of PD-1 inhibitors with conventional therapies has become a major advancement in the current clinical treatment of ESCC. Its core lies in enhancing efficacy through the synergistic interaction between immunotherapy and chemotherapy, radiotherapy, or targeted therapy.

In the first-line treatment of advanced ESCC, PD-1 inhibitor combined with chemotherapy significantly prolongs overall survival and PFS compared to chemotherapy alone, with a 32% reduction in the risk of death (HR = 0.68) and a 38% reduction in the risk of disease progression (HR = 0.62) (Jiao et al., 2025). Table 1 provides an overview of the key clinical trials discussed in this section. Additionally, the ORR is significantly improved (OR = 2.08) (Xu et al., 2018). The advantage of this combination strategy is particularly pronounced in patients with high PD-L1 expression (CPS ≥10) (Pfannenstiel et al., 2010; Kim et al., 2024). It is noteworthy that although combination therapy increases the risk of grade 3 or higher irAEs (OR = 3.70), the overall safety profile is manageable, and there is no statistically significant difference in grade 3–5 treatment-related adverse events (trAEs) compared to chemotherapy alone (Pfannenstiel et al., 2010; Duan et al., 2018).

**TABLE 1:** Pivotal Phase II/III Clinical Trials of PD-1/PD-L1 Inhibitor-based combination Therapy in ESCC. the data comes from (Qin et al., 2024; Sun et al., 2021; Wang Z. X. et al., 2022; Doki et al., 2022; Xu et al., 2025; Kelly et al., 2021).

Parameter	KEYNOTE-590	ESCORT-1st	JUPITER-06	CheckMate 648	RATIONALE-306	ORIENT-15	ESCORT-NEO	CheckMate 577	Parameter	KEYNOTE-590	ESCORT-1st	JUPITER-06
Phase	III	III	III	III	III	III	III	III	Phase	III	III	III
Treatment setting	First-line advanced/metastatic	First-line advanced/metastatic	First-line advanced/metastatic	First-line advanced/metastatic	First-line advanced/metastatic	First-line advanced/metastatic	Neoadjuvant (resectable)	Adjuvant (post-nCRT + surgery)	Treatment setting	First-line advanced/metastatic	First-line advanced/metastatic	First-line advanced/metastatic
Sample size	749	596	514	970	649	659	391	794	Sample size	749	596	514
Experimental arm	Pembrolizumab + CF	Camrelizumab + TP	Toripalimab + TP	Nivolumab + chemo; nivolumab + ipilimumab	Tislelizumab + ICC	Sintilimab + chemo	Camrelizumab + nab-TP; camrelizumab + TP	Nivolumab	Experimental arm	Pembrolizumab + CF	Camrelizumab + TP	Toripalimab + TP
Control arm	CF	Placebo + TP	Placebo + TP	Chemotherapy	Placebo + ICC	Placebo + chemo	TP	Placebo	Control arm	CF	Placebo + TP	Placebo + TP
Primary endpoint	OS, PFS	OS	OS, PFS	OS	OS	OS	pCR, EFS	DFS	Primary endpoint	OS, PFS	OS	OS, PFS
OS (HR, 95% CI)	HR 0.72 (0.62–0.84); 12.3 vs. 9.8 months	HR 0.70 (0.56–0.88); 15.3 vs. 12.0 months	HR 0.72 (0.58–0.88); 17.7 vs. 12.9 months	Nivo + chemo: HR 0.74 (0.60–0.91); Nivo + ipi: HR 0.78 (0.65–0.95)	HR 0.70 (0.57–0.85); 17.2 vs. 10.6 months	HR 0.63 (0.51–0.78)	—	—	OS (HR, 95% CI)	HR 0.72 (0.62–0.84); 12.3 vs. 9.8 months	HR 0.70 (0.56–0.88); 15.3 vs. 12.0 months	HR 0.72 (0.58–0.88); 17.7 vs. 12.9 months
PFS (HR, 95% CI)	HR 0.64 (0.54–0.75); 6.3 vs. 5.8 months	HR 0.56 (0.46–0.68); 6.9 vs. 5.6 months	Significant benefit reported in interim analysis	—	HR 0.62 (0.50–0.76)	HR 0.56 (0.46–0.68)	—	DFS: HR 0.69 (0.56–0.86); 22.4 vs. 11.0 months	PFS (HR, 95% CI)	HR 0.64 (0.54–0.75); 6.3 vs. 5.8 months	HR 0.56 (0.46–0.68); 6.9 vs. 5.6 months	Significant benefit reported in interim analysis

For locally advanced resectable ESCC, NICT (i.e., PD-1 inhibitor combined with chemotherapy followed by surgery) significantly improves the pCR rate and the major pathological response rate compared to traditional neoadjuvant chemotherapy (nCT), without increasing the risk of serious postoperative complications such as pneumonia or anastomotic leakage. This means more patients can achieve tumor downstaging, creating favorable conditions for radical surgery. Furthermore, studies have shown that although neoadjuvant immunotherapy may prolong the interval from the last treatment to surgery and increase the risk of anemia, it does not affect the R0 resection rate or the number of lymph nodes dissected (Isaacs and Stinchcombe, 2022).

Beyond combination with chemotherapy, PD-1 inhibitors are also combined with anti-angiogenic targeted drugs and radiotherapy. For example, PD-1 inhibitor combined with anlotinib (a multi-target anti-angiogenic TKI) has shown promising antitumor activity and manageable safety in later-line treatment for advanced ESCC progressing after chemotherapy. Another chemotherapy-free strategy, namely, PD-1 inhibitor combined with anlotinib as first-line treatment for advanced ESCC, has also shown encouraging preliminary efficacy (ORR reaching 60.9%) and tolerable adverse events in early studies (Huang et al., 2021).

However, the efficacy of PD-1 inhibitor-based combination therapy is heterogeneous. For instance, patients with PD-L1-negative (CPS<1) tumors and current smokers may not derive significant survival benefit from combination therapy (Jiao et al., 2025), Therefore, future research needs to further explore and optimize biomarkers to precisely identify patients who are more likely to benefit, and to investigate novel combination strategies to overcome resistance.

### 6.1 PD-1 inhibitors in combination with surgery

The combination of PD-1 inhibitors with surgery is a crucial strategy in the contemporary comprehensive treatment of ESCC. It integrates two primary modalities: preoperative neoadjuvant therapy and postoperative adjuvant therapy, aiming to maximize tumor eradication, reduce the risk of recurrence, and improve long-term patient survival.

NICT followed by surgery is currently the most extensively researched and widely applied model. Typically, 2–4 cycles of a PD-1 inhibitor (e.g., tislelizumab, sintilimab, pembrolizumab) combined with chemotherapy (commonly a regimen of nab-paclitaxel plus a platinum agent) are administered prior to surgery. The objectives are to downstage the tumor, increase the R0 resection rate (complete microscopic resection), and eliminate micrometastases, after which surgery is performed (McDermott and Atkins, 2013). For patients who did not receive neoadjuvant immunotherapy before surgery or who have high-risk postoperative features (such as positive surgical margins or lymph node involvement), adjuvant therapy with a PD-1 inhibitor monotherapy may be continued after surgery. This aims to further eliminate residual tumor cells and reduce the risk of recurrence.

Releasing Immune Suppression and Activating Systemic Anti-tumor Responses: PD-1 inhibitors block the interaction between PD-L1 on tumor cells and PD-1 on T cells, thereby removing the “brake” that the tumor microenvironment places on T cells. This reactivates the ability of cytotoxic T cells to recognize and attack cancer cells. Preoperative application creates a more favorable local and systemic environment for surgery. Improving Pathological Response Rates and Prognosis: Neoadjuvant immunotherapy can significantly increase rates of pCR and major pathological response (MPR). Patients who achieve pCR or MPR typically have superior long-term event-free survival (EFS) and overall survival (OS) (McDermott and Atkins, 2013). Synergistic Effects: Chemotherapeutic agents can promote tumor antigen release and ICD, thereby synergizing with PD-1 inhibitors to enhance anti-tumor immune responses (Alsaab et al., 2017). Modulating the TIME: TIME refers to the dynamic ecosystem of cellular and molecular components surrounding a tumor, including immune cells, stromal cells, vascular cells, the extracellular matrix, and various signaling molecules (Zou and Chen, 2008; Yi et al., 2021). The combination of surgery and immunotherapy may alter the tumor microenvironment, reducing immunosuppression and promoting immune cell infiltration.

A single-arm Phase II study evaluated the efficacy of tislelizumab combined with carboplatin and nab-paclitaxel as neoadjuvant therapy for resectable ESCC. The results showed that the 4-year OS rate reached 69.4% in the surgery group, significantly higher than the 33.3% in the non-surgery group, and patients who achieved R0 resection had a better prognosis (Yan et al., 2022). While the perioperative immunotherapy model (NICT followed by surgery and adjuvant immunotherapy) has shown promising results in other tumor types, particularly in non-small cell lung cancer (e.g., the CheckMate 77T trial (Brazel et al., 2024), these data should be considered as indirect supporting evidence rather than direct proof of efficacy in ESCC. Direct evidence specifically for ESCC is still emerging. To date, several Phase II/III trials have specifically evaluated NICT in ESCC and have reported encouraging pathological response rates, as discussed in detail below. In this review, we have prioritized ESCC-specific clinical data and have clearly identified non-ESCC studies as indirect evidence where referenced for contextual or mechanistic support. Research by Professor Ma Jianqun’s team at Harbin Medical University Cancer Hospital (Yang et al., 2024) confirmed that for patients with locally advanced ESCC, neoadjuvant therapy with the PD-1 inhibitor (camrelizumab) combined with chemotherapy is significantly effective and has a manageable safety profile. This study provides clinical evidence for the application of neoadjuvant immunotherapy combined with chemotherapy in EC. A Phase III study evaluated the efficacy and safety of perioperative toripalimab combined with neoadjuvant chemotherapy versus neoadjuvant chemotherapy alone for resectable locally advanced ESCC. The results showed that compared to chemotherapy alone, the combination group significantly increased the pCR rate and demonstrated a clear trend toward survival benefit. The safety of this combination therapy was manageable, with the most common irAEs being hypothyroidism. It did not significantly increase postoperative complications or grade 3–4 treatment-related adverse events (Lu et al., 2024). At the 2025 American Society of Clinical Oncology Gastrointestinal Cancers Symposium (ASCO GI), Professor Li Zhigang’s team from Shanghai Chest Hospital presented the results of a Phase II clinical study on neoadjuvant adebrelimab combined with chemotherapy for operable locally advanced ESCC (Wang Y. et al., 2025). The study indicated that with this immunocombination regimen, the pCR rate was as high as 38.9%, and safety was favorable. Adebrelimab, as a new-generation PD-L1 inhibitor independently developed in China, has previously shown significant efficacy in patients with extensive-stage small-cell lung cancer and was approved by China’s National Medical Products Administration for first-line treatment in 2023. This study offers new hope for ESCC patients, marking an important step in the transition from traditional chemotherapy to immunocombination chemotherapy, especially with potential advantages in improving pCR rates and long-term survival. Although primarily focused on mechanistic exploration, a study utilizing single-cell transcriptomics found that PD-1 mediates radiotherapy resistance in ESCC. Researchers innovatively developed pH-responsive oxygen-carrying nanobubbles coupled with nivolumab, confirming in preclinical models that it can specifically activate CD8^+^ T cells and improve the hypoxic tumor microenvironment, significantly enhancing the sensitivity to 125I particle radiotherapy. This provides new insights for future multi-modal combinations of PD-1 inhibitors with radiotherapy, surgery, and other approaches. Of course, when combining PD-1 inhibitors with surgery, it is necessary to pay attention to treatment-related adverse reactions such as rash, thyroid dysfunction, immune-mediated pneumonitis, colitis, as well as the inherent toxic side effects of chemotherapy. Although most adverse reactions are controllable, close monitoring and timely management are still required. Regarding the timing of surgery after neoadjuvant immunotherapy, the surgical timing requires careful assessment by a multidisciplinary team (MDT). Current evidence indicates that such treatment does not significantly increase surgical difficulty or serious postoperative complications (Figure 2).

**FIGURE 2:**
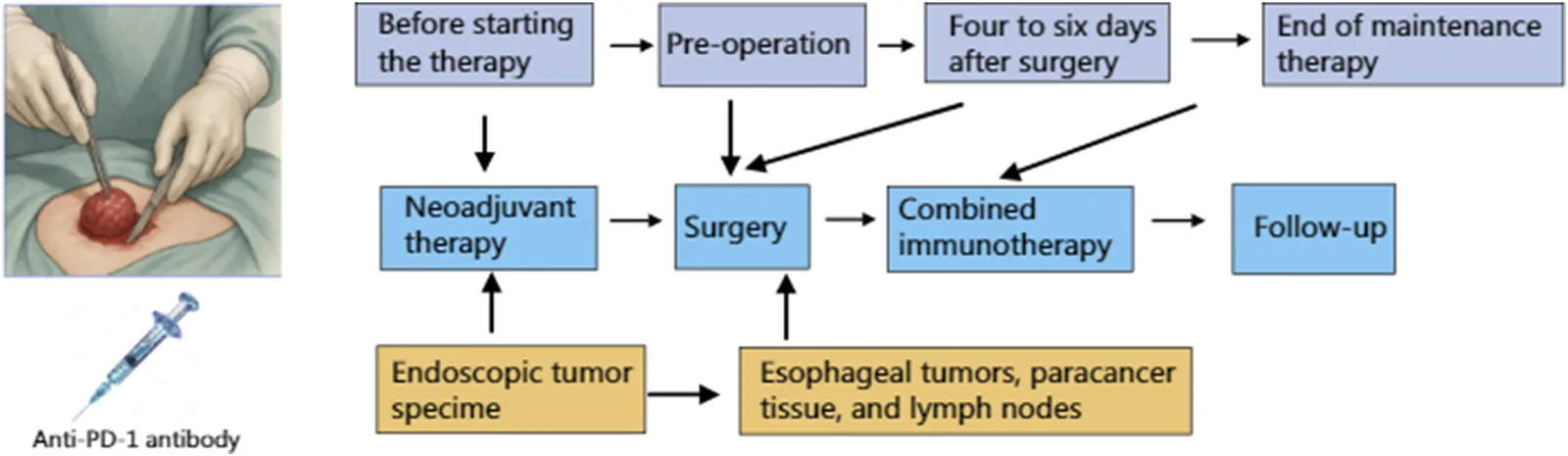
A summary of PD-1 inhibitors in combination with surgery(original).

### 6.2 PD-1 inhibitors in combination with radiotherapy

The combination of PD-1 inhibitors with radiotherapy represents a powerful comprehensive treatment strategy for ESCC with strong synergistic potential. Its core principle lies in integrating the systemic effects of immunotherapy with the local control achieved by radiotherapy, aiming for a greater-than-additive (“1 + 1>2″) therapeutic outcome. The primary combination modalities include concurrent radio-immunotherapy and sequential radio-immunotherapy.

The synergistic effect of combining PD-1 inhibitors with radiotherapy stems from multiple immunobiological mechanisms, including reversing T-cell exhaustion and activating anti-tumor immunity, the abscopal effect, remodeling the TIME, and ameliorating the hypoxic microenvironment to overcome radiotherapy resistance. Radiotherapy can induce ICD in tumor cells, releasing tumor-associated antigens and danger signal molecules that activate antigen-presenting cells, thereby initiating a T-cell response. However, the upregulated PD-1/PD-L1 pathway in the tumor microenvironment can lead to CD8^+^ T-cell exhaustion. PD-1 inhibitors block this pathway, releasing the “brake” on T cells and restoring their cytotoxic function (Alsaab et al., 2017). The abscopal effect is one of the most compelling mechanisms of this combined strategy. Local radiotherapy not only kills tumor cells within the irradiation field but may also trigger a systemic anti-tumor immune response through the released tumor antigens and inflammatory signals, leading to shrinkage of unirradiated distant metastases. PD-1 inhibitors can enhance and sustain this systemic effect (Yang and Ge, 2020). Furthermore, tumor hypoxia is a significant factor contributing to radiotherapy resistance. Improving tumor hypoxia while concurrently blocking PD-1 can significantly enhance the activation and tumor-killing function of CD8^+^ T cells, thereby increasing radiosensitivity (Doki et al., 2005).

A retrospective study investigated the efficacy and safety of adding radiotherapy (RT) to a PD-1 inhibitor as a second-line or later regimen for recurrent/metastatic advanced ESCC (Cancer Medicine, 2025). In the entire study population (169 patients), the median PFS and overall survival were numerically better in the radiotherapy combined with immunotherapy group (RT group) compared to the immunotherapy-alone group (non-RT group), although the difference did not reach statistical significance. This study suggests that for advanced ESCC patients receiving immunotherapy, especially those with local/regional recurrence, adding radiotherapy as a local treatment modality can significantly improve efficacy and survival outcomes with a manageable safety profile, providing an important reference for clinical practice.

Despite the promising prospects of combining PD-1 inhibitors with radiotherapy, several challenges remain. The combination may increase the risk of adverse events, particularly requiring close monitoring and timely intervention for overlapping toxicities like immune-mediated pneumonitis and radiation pneumonitis. Other common toxicities include radiation esophagitis, dermatitis, thyroid dysfunction, and colitis. Research indicates that while combination therapy may increase the incidence and severity of certain toxicities (e.g., radiation esophagitis, esophageal fistula), its overall safety profile is manageable.

In summary, the combination of PD-1 inhibitors with radiotherapy, through mechanisms such as the abscopal effect, reversal of T-cell exhaustion, remodeling of the tumor immune microenvironment, and overcoming resistance by improving hypoxia, provides a powerful treatment option for patients with locally advanced, recurrent, or metastatic ESCC. Clinical data, whether from prospective trials or real-world studies, support its favorable efficacy and manageable safety (Zhang et al., 2021) (Figure 3).

**FIGURE 3:**
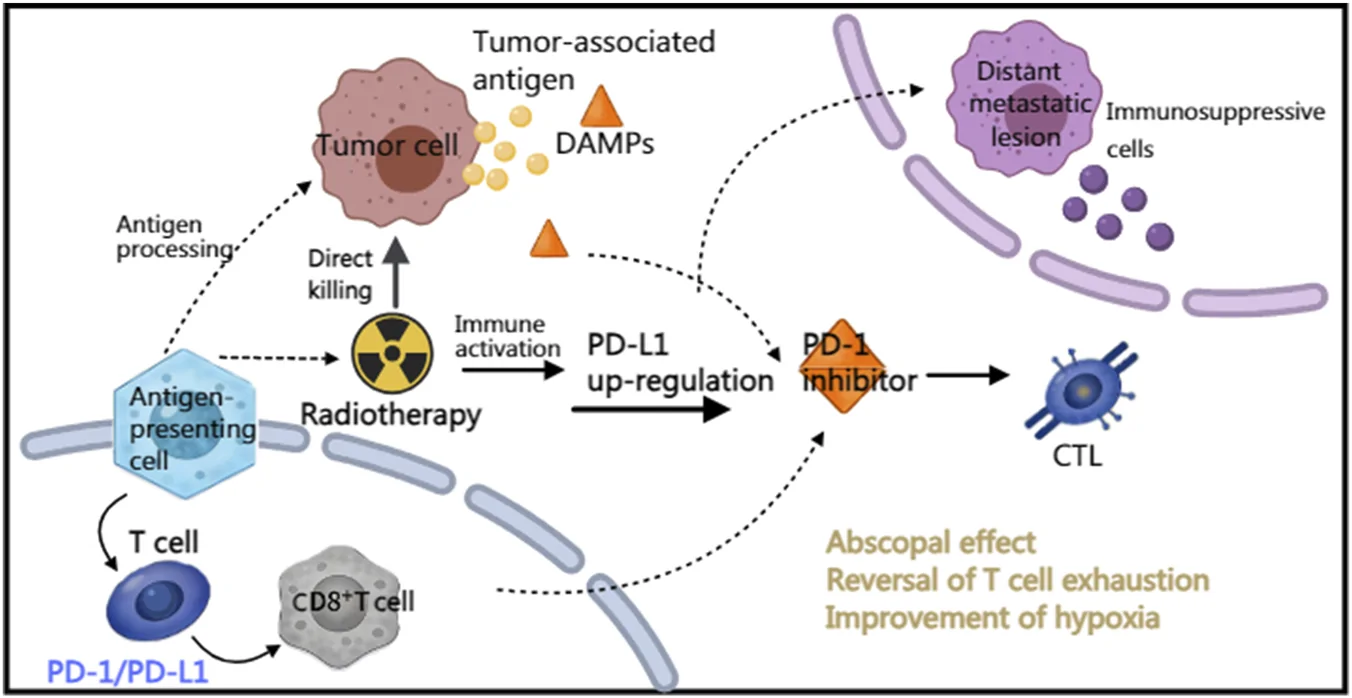
A summary of PD-1 inhibitors in combination with radiotherapy.

### 6.3 PD-1 inhibitors in combination with chemotherapy

The combination of PD-1 inhibitors with chemotherapy is a widely adopted and effective clinical strategy for ESCC, offering new hope, particularly for patients with advanced disease. For unresectable locally advanced, recurrent, or metastatic ESCC, PD-1 inhibitor combined with platinum-based doublet chemotherapy (e.g., paclitaxel + cisplatin/carboplatin, or 5-FU + cisplatin) has become the standard first-line treatment regimen (Jiaying et al., 2024). For resectable locally advanced ESCC, the preoperative use of PD-1 inhibitors combined with chemotherapy (NICT) aims to achieve tumor downstaging, improve R0 resection rates and pathological response rates, thereby creating favorable conditions for subsequent surgery.

The synergy primarily arises from several mechanisms. Chemotherapeutic agents, such as taxanes and platinum-based drugs, can induce ICD in tumor cells. This process exposes tumor-specific antigens and releases damage-associated molecular patterns (DAMPs), effectively tagging the targets for the immune system. This, in turn, activates antigen-presenting cells (e.g., dendritic cells) and initiates a T-cell-mediated immune response (Gebremeskel and Johnston, 2015). Furthermore, chemotherapy can help selectively deplete immunosuppressive cells, such as regulatory T cells (Tregs) and myeloid-derived suppressor cells (MDSCs), thereby alleviating the immunosuppressive state of the tumor microenvironment (TME). PD-1 inhibitors act by further blocking the PD-1/PD-L1 pathway, releasing the braking inhibition on T cells and enabling the reactivated T cells to restore their tumor-killing function. Additionally, some chemotherapeutic agents may upregulate the expression of PD-L1 on tumor cells, which could paradoxically enhance the targeting and therapeutic effect of PD-1 inhibitors. In essence, chemotherapy directly kills tumor cells and rapidly reduces the tumor burden, while the immune system activated by PD-1 inhibitors is responsible for clearing residual cells and maintaining long-term immune memory. This complementary action results in a synergistic cytotoxic effect.

A Phase III clinical trial established pembrolizumab combined with chemotherapy (5-FU + cisplatin) as a standard first-line treatment for advanced ESCC (Kato et al., 2019). The study showed that, among all patients, pembrolizumab plus chemotherapy significantly reduced the risk of death by 27% and extended median OS compared to chemotherapy alone. Concurrently, it reduced the risk of disease progression or death by 35%, with an improvement in median PFS (6.3 months vs. 5.8 months). Analysis of the Chinese subgroup indicated a potentially more pronounced survival benefit trend, with a 49% reduction in the risk of death. Five-year follow-up data presented at the 2024 ASCO GI conference revealed a 5-year OS rate of 10.6% in the combination group, more than triple that of the control group (3.0%). This signifies that a considerable proportion of patients achieved long-term survival, which was scarcely imaginable in the chemotherapy era.

The ESCORT-first trial, which investigated 596 previously untreated patients with advanced or metastatic ESCC, demonstrated that patients receiving camrelizumab plus chemotherapy had a 30% reduction in the risk of death compared to those receiving chemotherapy alone—a difference with high statistical significance (*P* < 0.0001) (Luo et al., 2021). The median OS was 13.5 months in the combination group versus 12.0 months in the control group. Furthermore, the risk of disease progression or death was reduced by 46%. The final analysis of the ESCORT-first trial proved that first-line camrelizumab combined with chemotherapy provides significant and clinically meaningful survival benefits, including longer OS, extended disease control, and a doubling of the long-term survival rate, with a manageable safety profile, firmly establishing it as a standard first-line treatment for this patient population (Luo et al., 2021). Another study, the JUPITER-06 trial, evaluated the efficacy and safety of toripalimab combined with TP chemotherapy (paclitaxel + cisplatin) versus placebo combined with TP chemotherapy in previously untreated patients with advanced or metastatic ESCC (Yamamoto and Kato, 2022),This randomized, double-blind, placebo-controlled Phase III trial involved patients receiving toripalimab or placebo combined with paclitaxel and cisplatin, followed by maintenance therapy with toripalimab or placebo until disease progression, unacceptable toxicity, or for up to 2 years. The results showed that toripalimab plus chemotherapy significantly reduced the risk of disease progression or death by 42% compared to chemotherapy alone. In summary, the JUPITER-06 trial confirmed that in the first-line treatment of advanced ESCC, toripalimab combined with chemotherapy significantly improves both PFS and OS compared to traditional chemotherapy, with a manageable safety profile, providing a new and more effective therapeutic option for patients.

The ICD induced by chemotherapeutic agents is a multi-step molecular process. Upon exposure to ICD-inducing chemotherapeutics (e.g., taxanes, platinum agents, and anthracyclines), dying tumor cells undergo a series of tightly regulated events that convert them into *in situ* vaccines. First, calreticulin (CRT) is translocated from the endoplasmic reticulum to the cell surface, serving as an “eat-me” signal that facilitates phagocytosis by dendritic cells (DCs). Second, high-mobility group box 1 (HMGB1) is released from the nucleus into the extracellular space, where it binds to toll-like receptor 4 (TLR4) on DCs, promoting antigen processing and cross-presentation. Third, adenosine triphosphate (ATP) is secreted into the TME, acting as a “find-me” signal that recruits DCs and other antigen-presenting cells via purinergic receptor P2RX7 signaling. Collectively, these damage-associated molecular patterns (DAMPs) orchestrate the maturation and activation of DCs, which subsequently prime tumor-specific CD8^+^ T-cell responses. This ICD-driven T-cell priming synergizes with PD-1 inhibitors, which prevent the exhaustion of these newly activated T cells within the immunosuppressive TME (Gebremeskel and Johnston, 2015) (Figure 4).

**FIGURE 4:**
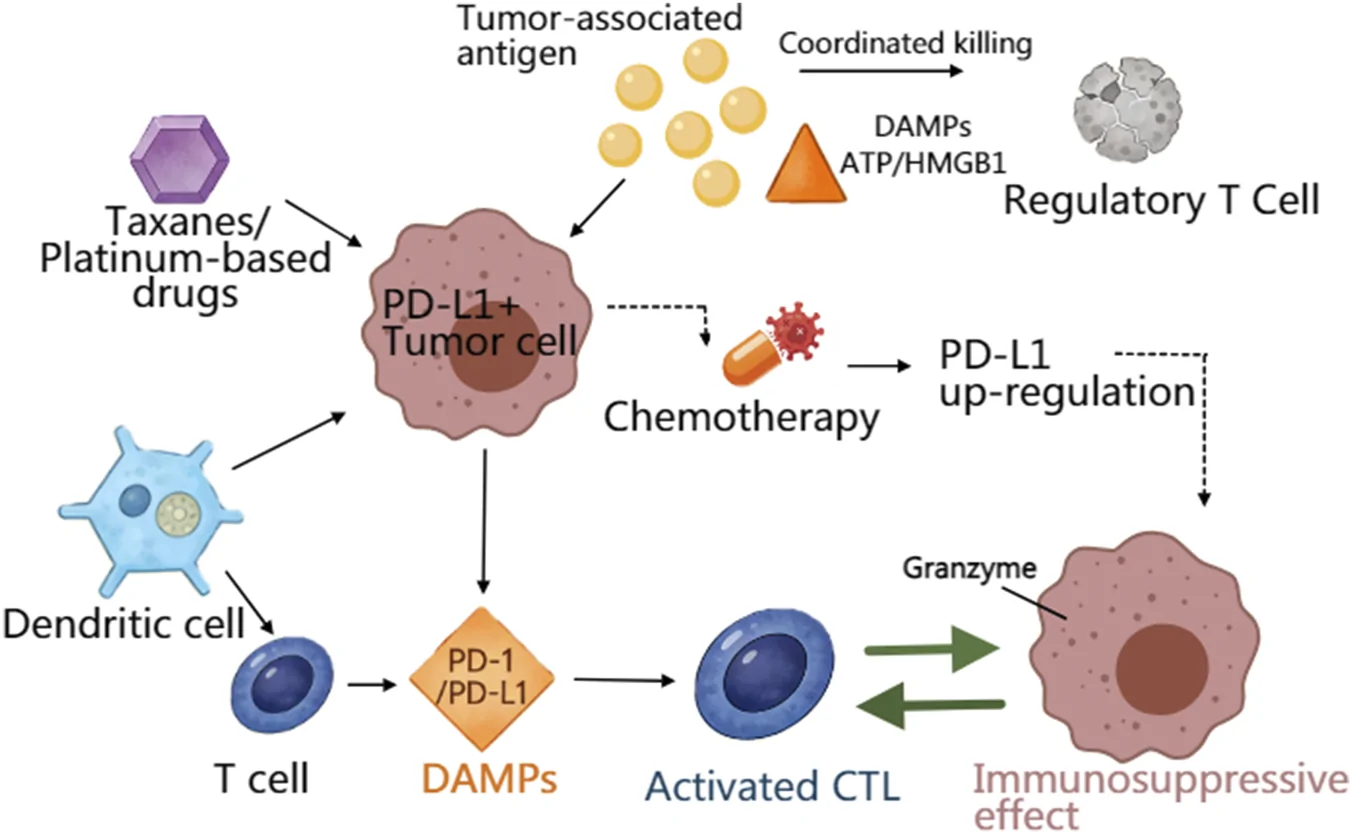
A summary of PD-1 inhibitors in combination with chemotherapy.

### 6.4 Biomarker-guided patient selection

Despite the robust efficacy of PD-1 inhibitor combination therapy in unselected ESCC populations, the magnitude of benefit varies substantially across patients. PD-L1 expression, assessed by CPS, is the most extensively validated biomarker, with patients harboring CPS ≥10 deriving greater survival benefits (Pfannenstiel et al., 2010; Kim et al., 2024). However, PD-L1-negative patients also derive some benefit, indicating that PD-L1 alone is insufficient as a sole selection criterion. Other candidate biomarkers—including TMB, circulating tumor DNA (ctDNA) dynamics, gut microbiome composition, and immune gene signatures—are under active investigation but have not yet been validated for clinical use in ESCC (Jiao et al., 2025). Future trials should incorporate biomarker-driven designs to identify the optimal candidates for combination therapy and potentially spare non-responders from unnecessary toxicity.

### 6.5 Mechanisms of primary and acquired resistance

While PD-1/PD-L1 blockade has demonstrated remarkable clinical activity, a substantial proportion of ESCC patients exhibit primary resistance (failure to respond to initial therapy) or acquired resistance (relapse after initial response). The mechanisms underlying resistance are multifactorial and include (1) defects in the IFN-γ signaling pathway (e.g., JAK1/2 mutations): that impair antigen presentation and T-cell trafficking; (2) loss of tumor antigen presentation due to downregulation of major histocompatibility complex (MHC) class I molecules or β2-microglobulin mutations; (3) upregulation of alternative immune checkpoints such as LAG-3, TIM-3, and TIGIT, which provide compensatory inhibitory signals; (4) accumulation of immunosuppressive cell populations, including MDSCs and Tregs, within the TME; and (5) tumor cell-intrinsic alterations, such as WNT/β-catenin pathway activation, that promote immune exclusion (Wang D. R. et al., 2022; Yu et al., 2026). A comprehensive understanding of these resistance mechanisms is essential for designing rational combination strategies to overcome them—a topic we discuss in greater detail in Section.

### 6.6 Toxicity management and safety considerations

The addition of PD-1 inhibitors to conventional therapies increases the risk of irAEs, including pneumonitis, colitis, hepatitis, dermatitis, and endocrinopathies (Xu et al., 2018). While most irAEs are manageable with corticosteroids and treatment interruption, severe or fatal irAEs (e.g., fulminant myocarditis, toxic epidermal necrolysis) require prompt recognition and aggressive management (Li M-Y. et al., 2025). Importantly, overlapping toxicities with chemotherapy (myelosuppression, neuropathy) and radiotherapy (radiation pneumonitis, esophagitis) pose additional challenges.

### 6.7 Special populations and unmet clinical needs

Beyond the general safety considerations discussed above, the applicability of PD-1 inhibitor combination therapy to specific patient subgroups remains inadequately characterized. Clinical trials of PD-1 inhibitor combinations in ESCC have predominantly enrolled patients aged <75 years with ECOG performance status 0–1 and without significant comorbidities. Consequently, evidence for the following common clinical scenarios remains limited.

Elderly patients (≥75 years): Age-related immunosenescence may alter both the efficacy and toxicity profile of PD-1 inhibitors (Zheng et al., 2025). While subgroup analyses from pivotal trials suggest that older patients derive similar relative benefits, absolute benefits may be attenuated by competing risks, and the risk of severe irAEs may be higher. Dedicated prospective studies in this population are lacking (Duan et al., 2024).

Patients with poor performance status (ECOG ≥2): These patients are systematically excluded from most registration trials, yet they represent a substantial proportion of real-world advanced ESCC cases (Sato et al., 2024). Retrospective data suggest that PD-1 inhibitors may still offer meaningful palliation in selected ECOG 2 patients, but the risk of treatment-related toxicity is heightened, and survival benefits are less pronounced than in fitter patients.

Patients with pre-existing autoimmune diseases (ADs): These patients have historically been excluded from immunotherapy trials due to concerns of flare of underlying Ads (Su et al., 2025). Emerging retrospective cohorts suggest that PD-1 inhibitors may be used with caution in select patients with well-controlled ADs, particularly those with mild, inactive disease, but the risk of AD flare (approximately 30%–50%) and severe irAEs is elevated compared to patients without ADs.

Future prospective trials should consider including these underrepresented populations to better define the risk-benefit profile of PD-1 inhibitor combination therapy in the full spectrum of ESCC patients encountered in routine clinical practice.

### 6.8 Optimal timing, duration, and sequencing of therapy

Several unanswered questions pertain to treatment logistics. The optimal timing of surgery after NICT remains controversial, with current practice relying on multidisciplinary team (MDT) assessment. The ideal duration of PD-1 inhibitor maintenance therapy—whether continued until progression, for a fixed duration (e.g., 1–2 years), or guided by ctDNA dynamics—has not been established in ESCC. Additionally, whether PD-1 inhibitors should be administered concurrently or sequentially with radiotherapy, and whether the same applies to chemotherapy, requires further investigation through dedicated comparative trials (Li Y. et al., 2025).

## 7 Conclusion

The combination of PD-1/PD-L1 inhibitors with conventional therapies (chemotherapy, radiotherapy, and surgery) demonstrates significant synergistic potential in the treatment of ESCC. This synergy is primarily evidenced by complementary and enhanced mechanisms of action as well as markedly improved clinical efficacy. This combined strategy not only boosts anti-tumor effectiveness but also provides more therapeutic options for patients across different disease stages. Through multi-mechanistic synergy, it has significantly improved the survival outcomes for ESCC patients, establishing itself as the standard first-line treatment for advanced ESCC and a crucial strategy in the perioperative setting.

The future requires further optimization of combination regimens, management of safety profiles, and exploration of precision treatment strategies to maximize clinical benefits for patients. Subsequent research is anticipated to continue investigating combinations of PD-1/PD-L1 inhibitors with other targeted agents, radiotherapy, and traditional Chinese medicine, aiming to benefit a broader patient population and even overcome resistance. Although combination therapy has shown clinically meaningful efficacy, it still faces numerous challenges. More basic and clinical research is needed to further advance the application of immunotherapy combined with conventional therapies.
